# Molecular characterization of partial fusion gene and C-terminus extension length of haemagglutinin-neuraminidase gene of recently isolated Newcastle disease virus isolates in Malaysia

**DOI:** 10.1186/1743-422X-7-183

**Published:** 2010-08-08

**Authors:** Ayalew Berhanu, Aini Ideris, Abdul R Omar, Mohd Hair Bejo

**Affiliations:** 1 Faculty of Veterinary Medicine, Universiti Putra Malaysia, 43400 UPM Serdang, Selangor, Darul Ehsan, Malaysia; 2 Faculty of Veterinary Medicine, University of Gondar, P.O. Box 176, Gondar, Ethiopia; 3 Institute of Bioscience, Universiti Putra Malaysia, 43400 UPM Serdang, Selangor Darul Ehsan, Malaysia

## Abstract

**Background:**

Newcastle disease (ND), caused by Newcastle disease virus (NDV), is a highly contagious disease of birds and has been one of the major causes of economic losses in the poultry industry. Despite routine vaccination programs, sporadic cases have occasionally occurred in the country and remain a constant threat to commercial poultry. Hence, the present study was aimed to characterize NDV isolates obtained from clinical cases in various locations of Malaysia between 2004 and 2007 based on sequence and phylogenetic analysis of partial F gene and C-terminus extension length of HN gene.

**Results:**

The coding region of eleven NDV isolates fusion (F) gene and carboxyl terminal region of haemagglutinin-neuraminidase (HN) gene including extensions were amplified by reverse transcriptase PCR and directly sequenced. All the isolates have shown to have non-synonymous to synonymous base substitution rate ranging between 0.081 - 0.264 demonstrating presence of negative selection. Analysis based on F gene showed the characterized isolates possess three different types of protease cleavage site motifs; namely ^112^RRQKRF^117^, ^112^RRRKRF^117 ^and ^112^GRQGRL^117 ^and appear to show maximum identities with isolates in the region such as cockatoo/14698/90 (Indonesia), Ch/2000 (China), local isolate AF2240 indicating the high similarity of isolates circulating in the South East Asian countries. Meanwhile, one of the isolates resembles commonly used lentogenic vaccine strains. On further characterization of the HN gene, Malaysian isolates had C-terminus extensions of 0, 6 and 11 amino acids. Analysis of the phylogenetic tree revealed that the existence of three genetic groups; namely, genotype II, VII and VIII.

**Conclusions:**

The study concluded that the occurrence of three types of NDV genotypes and presence of varied carboxyl terminus extension lengths among Malaysian isolates incriminated for sporadic cases.

## Background

Newcastle disease (ND) is a highly contagious disease of birds and has been regarded throughout the world as one of the most important diseases of poultry and other birds [1], in which infection with the extremely virulent viruses may result in sudden, high mortality with comparatively few clinical signs. The causative agent, NDV, is avian Paramyxovirus under the Avulavirus and has a negative-sense, single-stranded RNA genome [2]. So far, NDV strains with genomic sizes of 15,186, 15192 and 15198 nucleotides which codes for at least six proteins including nucleoprotein (N), phosphoprotein (P), matrix (M) protein, fusion (F) protein, haemagglutinin-neuraminidase (HN) protein and RNA polymerase (L) [2-4] have been identified. Among the six major proteins, the two interactive surface glycoproteins, the F and the HN proteins, are involved in cell surface attachment and cell membrane fusion [3,5].

The molecular basis for NDV pathogenicity has been shown to be dependent on the F protein cleavage site amino acid sequence which is clearly described by OIE [6] molecular definition of virulent NDV stating that any AMV-1 virus that has three basic amino acids, either lysine (K) or arginine (R), at the fusion protein cleavage site between residues 113 and 116 at the C-terminus of the F2, as well as phenylalanine at residue 117 of F1 and carboxyl terminus amino acid extension length which varies due to the varying location of termination codons within the HN protein, resulting in the expression of HN proteins with varying amino acid lengths. A longer reading frame consisting of HN0 precursor of 616 amino acid residue is expressed only by avirulent NDV strains and biologically active HN proteins of 571 and 577 amino acid residues are expressed by virulent and lentogenic viruses, respectively [7].

Three different NDV genotypes, II, III, and IV, were involved in the first panzootic of ND and were restricted to the specific geographic region; South East Asia in which the outbreak began. In the late 1960 s, NDV genotypes V and VI emerged and caused the second and third panzootics, respectively. After that, two novel NDV genotypes, VII and VIII, were found in Asia, Southern Africa, and a number of European countries [8-12]. Genotype VII was mainly responsible for recent outbreaks in the neighbouring countries of Taiwan and China [8,13-15] constituting the fourth panzootic of NDV.

Intensive vaccine programs have been implemented in Malaysia, but ND outbreaks and sporadic cases have occasionally occurred, even in well-vaccinated farms. A major epidemic of ND has occurred in Peninsular Malaysia from 2000-2001 peaking with 84 outbreaks and 525981 cases in 2001 [16] which cause substantial losses. Isolates of low virulence, HitchnerB1 and LaSota are the most common type of vaccines being used in the world including Malaysia. Other vaccines used in Malaysia include S, Ulster 2C, NDV-6/10 and enteric vaccine strain VG-GA [17]. Despite intensive vaccination programs with live vaccines, NDV remains a constant threat to the commercial poultry. Here, we describe molecular characterization of F and C-terminus extension length of HN protein genes of recently isolated Malaysian isolates and their phylogenetic relationship of among NDV isolates derived from other countries or regions. Thus, characterization of these recent isolates may help to gain invaluable information about the pathogenicity and epidemiological relationships.

## Results

### Nucleotide and predicted amino acid sequence analysis

Sequence analysis of a region between nucleotide positions 47-435 of F gene, encompassing proteolytic cleavage site of F0 protein revealed 18-55 nucleotide and 4-21 amino acid substitutions in Malaysian isolates and the calculation of synonymous and non-synonymous substitution rate demonstrate that all isolates have a rate ranging between 0.081 - 0.264, which is a value less than one. As illustrated in Table 1, in all the NDV isolates examined, the rate of synonymous substitution (0.126 - 0.306) dominated the rate of non-synonymous substitution (0.014 - 0.032). The maximum amino acid substitution was observed at amino acid position 11 compared to consensus in which, 6 isolates (MB043/06, MB091/05, MB093/05, MB095/05, MB128/04 and MB85/05) had threonine (T) residue, and 3 isolates (MB016/07, MB064/05, MB061/06) had an alanine (A) residue. Isolates MB091/05, MB093/05, MB095/05 and MB128/04 shared unique amino acid substitutions with MB043/06 at amino acid position 13; proline (P) for leucine (L), C25F, T29A, K78R and Q114R (Table 2 and Table 3). The unique amino acid substitution suggests that these isolates might have a common origin despite being isolated in different areas in Malaysia. As shown in Table 4, the deduced amino acid sequence of the F0 protein cleavage site revealed that isolates MB043/06, MB091/05, MB093/05, MB095/05, and MB128/04 had an arginine (R) residue at position 114, which result in an ^112^RRRKRF^117 ^motif and the remaining isolates MB047/05, MB064/05, MB076/05 and MB085/05) carry the residue ^112^RRQKRF^117^. Only one isolate (MB016/07) possessed the motif of ^112^GRQGRL^117 ^common to avirulent isolates. Comparisons of nucleotide identities with published local and foreign isolates indicated that seven isolates (MB076/05, MB047/05, MB043/06, MB091/05, MB093/05, MB095/05 and MB128/04) were found to have maximum identity (91.2% to 96.4%) with Indonesian isolate (Cockatoo/Indonesia/14698/90), two isolates (MB064/05 and MB016/07) with strain Ch/2000 from China (94.8 to 96.6%) and the remaining isolates, MB085/05 and MB061/06 with the local Malaysian isolate AF2240 (97.4%) and vaccine strain LaSota (98.8%), respectively (data not shown). These findings indicated the high similarity of isolates circulating in the South East Asian countries but still having limited variation with isolates from different geographical areas.

**Table 1:** Number of nucleotide and amino acid substitutions and Ka/Ks ratio of in the current Malaysian NDV isolates.

NDV Isolates	No. of nucleotide substitution*	No. of amino acid substitution*	Non-synonymous substitution rate (*K*A)	Synonymous substitution rate (*K*S)	Ratio *(K*A/*K*S)
MB061/06	55	21	0.081	0.306	0.265
MB043/06	37	8	0.030	0.276	0.109
MB016/07	29	9	0.028	0.202	0.139
MB064/05	22	5	0.014	0.173	0.081
MB076/05	24	8	0.032	0.144	0.222
MB047/05	18	4	0.018	0.126	0.143
MB091/05	34	9	0.032	0.242	0.132
MB093/05	34	9	0.032	0.242	0.132
MB095/05	35	9	0.032	0.252	0.127
MB128/04	34	9	0.032	0.242	0.132
MB085/05	18	4	0.014	0.134	0.104

* compared to the consensus nucleotide and amino acid sequences.

**Table 2:** Amino acid residue substitution of F gene for NDV strains of different genotypes.

Isolate/genotype/subgenotype	11	12	13	14	16	17	18	19	20	22	25	26	28	29	30	36
	
Consensus	V	P	L	M	I	T	R	I	M	I	C	I	L	T	S	P
MB061/06II	**A**	.	M	.	T	I	.	V	A	V	.	.	P	A	N	.
MB043/06VII	**T**	.	**P**	.	.	.	.	.	.	.	**F**	.	.	**A**	.	.
MB016/07 VII	**A**	.	.	.	.	I	.	.	.	.	**Y**	V	.	.	.	.
MB064/05VII	**A**	.	.	.	.	.	.	.	.	.	.	.	.	.	.	.
MB076/05VII	.	.	.	I	T	.	.	.	.	.	.	.	.	.	.	S
MB047/05VII	.	.	.	.	.	.	.	.	.	.	.	.	.	.	.	.
MB091/05 VII	**T**	**S**	**P**	.	.	.	**Q**	.	.	.	**F**	.	.	**A**	.	.
MB093/05 VII	**T**	**S**	**P**	.	.	.	**Q**	.	.	.	**F**	.	.	**A**	.	.
MB095/05 VII	**T**	**S**	**P**	.	.	.	**Q**	.	.	.	**F**	.	.	**A**	.	.
MB128/04 VII	**T**	**S**	**P**	.	.	.	**Q**	.	.	.	**F**	.	.	**A**	.	.
MB085/05 VIII	**T**	.	.	.	.	.	.	.	**T**	.	.	.	.	.	.	.
AF2240 VIII	T	.	.	.	.	.	.	.	T	.	.	.	P	.	.	.
QH-1/79 VIII	T	.	S	.	.	.	.	.	.	.	.	.	.	.	G	.
QH-4/85VIII	T	.	S	.	.	.	.	.	.	.	.	.	.	.	G	.
V4 Queensland I	.	.	.	.	T	V	.	V	.	A	.	V	P	.	.	.
Ulster/67 I	.	.	.	.	T	V	.	V	A	E	.	V	P	.	.	.
LaSota II	A	.	M	T	T	I	.	V	A	V	.	.	P	A	N	.
Miyadera III	A	.	.	.	T	I	W	.	A	A	.	V	.	.	.	.
Herts/33 IV	A	.	P	.	.	I	.	.	V	T	.	.	.	.	.	.
Italien IV	.	.	.	.	.	I	.	.	A	T	.	.	.	.	.	.
CA1085/71 V	.	.	.	.	.	.	.	.	T	.	.	.	.	.	.	.
H-10/72 V	.	.	.	.	.	.	.	.	T	.	.	.	.	.	.	.
Iraq AG68 VI	.	.	.	.	.	.	.	.	.	.	.	.	.	.	.	.
Lebanon -70 VI	.	.	.	.	.	.	.	.	.	.	.	.	.	.	.	.
TX3503/04 VI	A	.	.	.	.	.	.	.	T	.	.	.	.	.	.	.
NDV05-027VI	A	.	.	.	.	.	.	.	T	V	.	.	.	.	.	.
Q-GB 506/97VI	P	.	.	.	.	.	.	.	V	.	S	V	.	.	.	.
DK-1/95VI	.	.	P	.	.	.	.	.	T	.	.	.	.		.	.
DE 143/95VII	.	.	.	.	.	.	.	.	.	.	.	.	.	.	.	.
Cockatoo/14698/90VII	.	.	.	.	.	.	.	.	.	.	.	.	S	.	.	.
ZA360/95VII	.	.	.	.	.	.	.	V	.	.	.	.	.	.	G	.
ZW 3422/95VII	.	.	.	.	.	.	.	V	.	.	.	.	.	.	G	.
NDV05-055 VII	A	.	.	I	.	.	.	.	.		.	.	.	A	.	.
Ch/2000VII	A	.	.	.	.	.	.	.	.	.	.	.	P	.	.	.
TW/2000 VII	A	.	.	T	.	.	.	.	.	.	.	.	.	.	.	.
TW/95-1VII	.	.	.	.	.	.	.	.	.	.	.	.	.	.	.	.
ZhJ-1/85	A	.	.	.	T	A	.	.	A	A	.	V	.	.	N	.
FJ-1/85	A	.	.	.	T	V	.	.	A	A	.	V	.	.	N	.

Genetic identity with consensus sequence is marked by dot (.).The bolded residues were residues discussed in the text.

**Table 3:** Amino acid residue substitution of F gene for NDV strains of different genotypes.

Isolate/genotype/Subgenotype	52	71	78	79	82	101	104	107	108	114	115	117	121	124
	
Consensus	I	K	K	A	E	R	G	S	T	Q	K	F	I	S
MB061/06 II	.	.	.	.	D	.	E	T	.	.	G	L	.	G
MB043/06 VII	.	.	**R**	.	.	**K**	.	.	M	**R**	.	.	.	.
MB016/07 VII	V	R	.	.	.	K	.	.	.	.	.	.	V	.
MB064/05 VII	V	R	.	.	.	K	.	.	.	.	.	.	V	.
MB076/05 VII	.	.	.	.	.	.	.	.	.	.	.	.	V	.
MB047/05 VII	.	.	.	.	.	K	.	A	.	.	.	.	V	.
MB091/05 VII	.	.	**R**	.	.	**K**	.	.	.	**R**	.	.	.	.
MB093/05 VII	.	.	**R**	.	.	**K**	.	.	.	**R**	.	.	.	.
MB095/05 VII	.	.	**R**	.	.	**K**	.	.	.	**R**	.	.	.	.
MB128/04 VII	.	.	**R**	.	.	**K**	.	.	.	**R**	.	.	.	.
MB085/05 VIII	.	.	.	**T**	.	.	.	**T**	.	.	.	.	.	.
AF2240 VIII	.	.	.	P	.	.	.	T	.	.	.	.	V	.
QH-1/79 VIII	.	.	R	T	.	.	.	T	.	.	.	.	V	.
QH-4/85 VIII	.	.	R	T	.	.	.	T	.	.	.	.	V	.
V4 Queensland I	.	.	.	.	.	.	E	T	.	.	G	L	.	G
Ulster/67 I	.	.	.	.	.	.	E	T	.	.	G	L	.	G
LaSota II	.	.	.	.	D	.	E	T	.	.	G	L	.	G
Miyadera III	.	.	.	.	.	.	E	T	.	.	R	.	.	.
Herts/33 IV	.	.	.	.	.	.	E	T	.	.	R	.	.	.
Italien IV	.	.	.	.	.	.	E	T	.	.	R			
CA1085/71 V	.	.	.	.	.	.	.	T	.	.	.	.	.	.
H-10/72 V	.	.	.	.	.	.	.	T	.	.	.	.	.	.
Iraq AG68 VIa	.	.	.	.	.	.	.	.	.	.	.	.	.	.
Lebanon -70 VIa	.	.	.	.	.	.	.	.	.	.	.	.	.	.
TX3503/04 VIb	.	.	.	.	.	.	.	.	.	K	.	.	.	.
NDV05-027VIb	.	.	.	.	.	.	.	.	.	.	.	.	.	.
Q-GB 506/97VIc	.	R	.	.		.	.	.	.	.	.	.	.	.
DK-1/95VId	.	.	.	.	.	.	.	.	.	.	.	.	.	.
DE 143/95VIIa	.	.	.	.	.	K	.	.	.	.	.	.	V	.
Cockatoo/14698/90VIIa	.	.	.	.	.	K	.	.	.	.	.	.	V	.
ZA360/95VIIb	.	.	.	.	.	.	.	.	.	.	.	.	V	.
ZW 3422/95VIIb	.	.	.	.	.	.	.	.	.	.	.	.	V	.
NDV05-055 VIIc	.	.	.	.	.	K	.	.	.	.	.	.	V	.
Ch/2000VIId	V	R	.	.	.	K	.	.	.	.	.	.	V	.
TW/2000 VIId	V	.	.	.	.	K	.	.	.	.	.	.	V	.
TW/95-1VIIe	.	.	.	T	.	K	.	.	.	.	.	.	V	.
ZhJ-1/85 IX	.	.	.	.	.	.	E	T	.	.	.	.	.	.
FJ-1/85 IX	.	.	.	.	.	.	E	T	.	.	.	.	.	.

**Table 4:** List of Newcastle disease virus isolates collected in Malaysia between 2004 and 2007 and their deduced amino acid sequence at the cleavage site.

Isolate ^ɸ^	Date of collection (DD/MM/YY)	F protein cleavage site*	Accession No. (F gene)	Origin
MB061/06	15/11/06	^112^GRQGR↓L^117^	GQ901891	Selangor
MB043/06	17/10/06	^112^RRRKR↓F^117^	GQ901896	Selangor
MB016/07	14/06/07	^112^RRQKR↓F^117^	GQ901894	Melaka
MB064/05	24/8/05	^112^RRQKR↓F^117^	GQ901893	Selangor
MB076/05	7/10/05	^112^RRQKR↓F^117^	GQ901892	Sabah
MB047/05	5/07/05	^112^RRQKR↓F^117^	GQ901895	Selangor
MB091/05	22/11/05	^112^RRRKR↓F^117^	GQ901897	Sabah
MB093/05	6/12/05	^112^RRRKR↓F^117^	GQ901898	Sabah
MB095/05	6/12/05	^112^RRRKR↓F^117^	GQ901899	Sabah
MB128/04	29/12/04	^112^RRRKR↓F^117^	GQ901900	Selangor
MB085/05	22/11/05	^112^RRQKR↓F^117^	GQ901901	Sabah

ɸ All the isolates were isolated from chicken except for MB061/06 isolated from parrot.

* Numbers indicate amino acid positions.

### Carboxyl terminus of HN protein gene

Nucleotide sequencing and subsequent deduction of the amino acid sequence covering the C- terminus of the HN protein revealed differences in HN length of amino acid sequences. Isolates MB016/07, MB043/06, MB047/05, MB064/05, MB076/05, MB091/05, MB093/05, MB095/05, and MB128/04 had no amino acid extension length with a total HN length of 571 amino acids regardless of their cleavage site sequence profiles, whereas isolates MB085/05 and MB061/06 had HN C-terminus extension length of 11 aa and 6 aa, respectively, as shown in Table 5. Isolate MB085/05 had the same C-terminus aa extension length and composition as that of the local isolate AF2240 while isolate MB061/06 revealed similar aa and C-terminus extension length with that of common vaccine strains such as LaSota, VG/GA, and Herts/33 used in Malaysia.

**Table 5:** Deduced HN protein amino acid sequence and C-terminus extension lengths of Malaysian NDV isolates.

NDV isolate	Deduced amino acid sequence	C-terminus amino acid extension length**	HN***	Accession No.
MB061/06	KDDGVREARSG*	6	577	GQ922495
MB043/06	KDNRA*	0	571	GQ922500
MB016/07	KDDRV*	0	571	GQ922498
MB064/05	KDDRV*	0	571	GQ922497
MB076/05	KDDRV*	0	571	GQ922496
MB047/05	KDDRV*	0	571	GQ922499
MB091/05	KDDRV*	0	571	GQ922501
MB093/05	KDDRV*	0	571	GQ922502
MB095/05	KDDRV*	0	571	GQ922503
MB128/04	KDDRV*	0	571	GQ922504
MB085/05	KDDRLQEVRSGRLSQP*	11	581	GQ922505

*Indicates the stop codon

**No C-terminus amino acid extension length was detected in all the isolates except for MB061/06 and MB085/05 with 6 and 11 amino acid extension lengths, respectively.

***Predicted length of the HN protein in number of amino acids based on ORF analysis of the gene nucleotide sequence.

### Phylogenetic analysis

**Figure 1:**
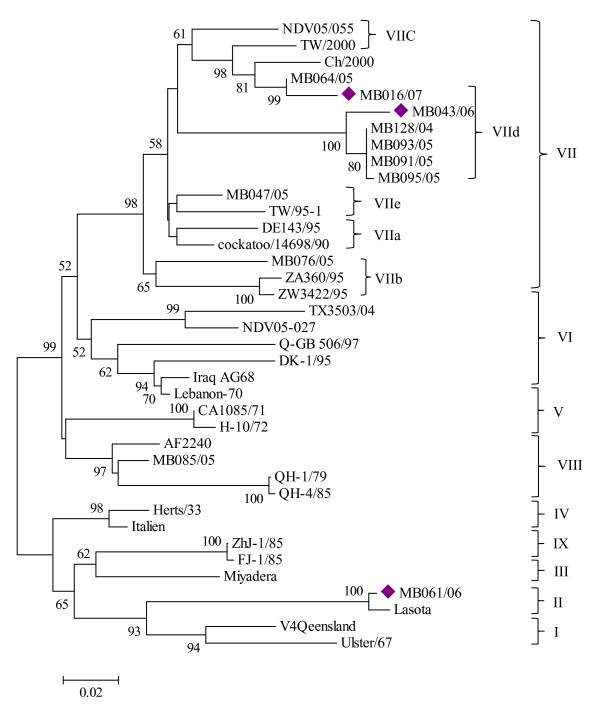
**Phylogenetic relationship among 38 NDV isolates on basis of F gene nucleotide sequences between position 47 and 435**. Sequences were obtained either from the present study or GenBank. The phylogenetic tree was constructed using neighbour- joining method on MEGA 4. Malaysian isolates in the current study were indicated by the rotated black square.

Phylogenetic tree generated based on the variable portion of the F gene between nt 47 - 435 of the 3 isolates and other 35 local and foreign NDV isolates worldwide separated into 9 potential clusters corresponding to the different genotypes of NDV. All the Malaysian isolates formed six major clusters despite their geographical entity and within these, the presence of viruses belonging to three of the nine genotypes were identified (Figure 1). Isolates MB016/07and MB043/06 characterized in this study were assigned to genotype VII within they belonged to previously established subgenotype VIId (Figure 1). Isolate MB043/06 together with other five local strains segregated into a monophyletic group with 100% bootstrap value within genotype VII as shown in Figure 1. Considering the topology of the phylogenetic tree, these Malaysian isolates might have a common origin

Previous phylogenetic analysis performed based on variable region of F gene at nt 206-421(216 bp) classified isolate MB085/05 as genotype VII [18]. In this study, however, it was grouped together with previously characterized local NDV isolate AF2240 (isolated in the 1960s) and Chinese isolates (QH-1/79 and QH-4/85) in genotype VIII. The phylogenetic grouping of MB085/05 as genotype VIII is supported by unique V^11^→T, A^79^→T and S^107^→T substitutions although isolates grouped into genotype VII in previous studies lacked the aforementioned characteristic (Table 2 and Table 3). The phylogenetic tree also showed that the lentogenic isolate (MB061/06) was closely related to strain LaSota.

## Discussion

Analysis of the ratio of synonymous and non-synonymous substitution rate in the current isolates demonstrated the presence of only purifying (negative) selection, despite the most variable portion of the F gene between nucleotide positions 47-435 was used in the analysis. Similar results were also observed previously on analysis of other entire NDV genes such as L (*K*a/*K*s = 0.064), M (*K*a/*K*s = 0.14 and P *(K*a/*K*s = 0.25) genes [19-21] which corresponds to negative selection.

The amino acid sequence of the protease cleavage site reveals that all of the isolates except isolate MB061/06 maintained multiple basic amino acids motifs within the penta-amino acid sequence of the F0 cleavage signal between residues 113 and 116 and phenylalanine (F) on the residue 117 on the N-terminus. It is widely accepted that the number of basic amino acids immediately upstream to the F0 protein cleavage site determines viral pathogenicity which is clearly described by OIE [6]. The presence of these characteristic patterns of amino acid demonstrated that the isolates could be considered as virulent except isolate MB061/06. It is a paramount importance to note that, the F0 cleavage site of isolate MB043/06 isolated from Selangor, Peninsular Malaysia, is unusual, containing an arginine (R) for glutamine (Q) substitution at residue 114. In our previous studies, isolates MB091/05, MB093/05, MB095/05, and MB128/04 from different districts of Sabah, West Malaysia showed similar F0 cleavage site characteristics [18]. However, in recent years, emergence of similar results were reported, in South African genotype VIII viruses [11], in Taiwan [15] and from Eurasian collard dove and pigeons isolates containing a ^112^R-R-K-K-R^116 ^, ^112^R-R-Q-K-R^116^, and ^112^R-R-R-K-R^116 ^motif [22-26]. Even though the contribution of arginine (R) at amino acid 114 in our isolates needs further studies, other studies indicated that arginine residue at different positions 113, 115 and 116 contribute for intracellular cleavage of virulent NDV fusion proteins [27].

Analysis of C-terminus extension length of HN protein gene revealed that the nine virulent NDV isolates shared 0 amino acid extension length with a total HN length of 571 amino acids regardless of their cleavage site sequence profiles (terminating in the sequence KDDRV and KDNRA), except MB085/05. In the present study, isolate MB085/05 was shown to have 11aa HN C-terminus extension length. This might have been due to a point mutations occurred at position 8164 (T→C) replacing the stop codon ^8164^TAA^8166 ^with ^8164^CAA^8166 ^codon and hence delayed terminations. This result may suggest a genetic relationship of MB085/05 sequences to the local virulent isolates with a genetic evolution that has led to the presence of these varied HN C- terminus extension lengths. In contrast, MB061/06 isolated from parrot was shown to have 6 amino acid C-terminus extension lengths, similar to that of common vaccine strains such as LaSota, VG/GA, and Herts/33 used in Malaysia. However, it is shorter than other avirulent strains such as Queensland V4 strain and Ulster/67, most of which have 45 amino acid extensions. Substitutions at positions 8149 (C→G) and 8150 (G→A) might have contributed for generation of the stop codon ^8149^TAG^8151^, terminating early and hence fewer amino acid extensions. It has been indicated that the shortest amino acid value (571 amino acids) was found in genotypes III-VIII consisting exclusively of viscerotropic velogenic strains [4]. The phylogenetic relationships based on the partial sequence of F protein gene of these isolates also showed that all the nine NDV isolates with shortest amino acid value (571) appeared to be closely related to viruses from genotype VII. According to Gould et al [28] and Kattenbelt et al [29], Australian isolates have HN extensions of 7, 9, 11, 14 or 45 amino acids and they indicated that viruses with 7 or 14 amino acid extensions were shown to be associated with summer respiratory disease.

The phylogenetic relationship result showed that the 3 Malaysian isolates recovered in 2006 and 2007; belong to genotype II and VII. Closer phylogenetic relationship of genotype VII viruses suggests that isolate MB043/06 and local viruses isolated from 2004-2005 belong to a subgenotype VIId with 100% bootstrap value, all of the members of which encodes the ^112^RRRKRF^117 ^motif at the fusion cleavage site (Figure 1). In addition, we note that all those isolates share 6 common unique substitutions that distinguished them from all other subgenotypes/genotypes. According to Yu et al [30], genotype VIIb viruses evolved from genotype VIb viruses by producing a VII specific V121 for I substitution and then changed to VIIa and VIIc by producing a K101-for-R substitution and became VIId by producing additional V52-for-I and Y314-for-F substitutions. Exceptions were observed in the substitution of V at amino acid position 121 in our isolate but it did share the K substitution at amino acid residue 101 in which both K101 and V121 were unique features of genotype VII. Lien et al. [31] reports that Taiwanese subgenotype VIId viruses collected between 2003 and 2006 contained K^71 ^and V^52 ^in which similar features were observed in isolate MB016/07. Thus it is predicted that isolate MB016/07 can also be grouped under subgenotype VIId, which represents the most newly emerging NDV strains frequently occurring in the region especially from China and Taiwan. These findings supported by topology of phylogenetic tree suggest that it genetically related to genotype VII viruses but with the distinguishable subcluster.

Our study has also indicated the occurrence of other sub-genotype/genotype; VIIb and VIII viruses. Malaysian subgenotype VIIb virus in this study would have been more closely related to local and South East Asian countries' isolates because of their regional proximity. However, it has shared high sequence identities (91.7 - 92.5%) with Southern Africa subgenotype VIIb viruses than other Malaysian genotype VII viruses (87.4 - 90.4%). Previous studies also proposed that subgenotype VIIb strains isolated in Southern Africa might have originated in the Far East as their lack of diversity indicated that they cannot be indigenous to South Africa [8]. The finding in this work revealed that Malaysian genotype VII isolates are also phylogenetically related and share a common ancestor (supported by a high bootstrap value of 98) with Southern African isolates strengthening the hypothesis that genotype VII viruses were endemic in South East Asian countries. It has been documented that genotype VIII viruses were isolated from ND outbreaks in western China between 1979 and 1985, Southern Africa and are endemic in Southern Africa [8,11,13]. The presence of genotype VIII virus neighbouring country Singapore in early 1960 s and in Malaysia (AF2240) that gave rise to the recent genotype VIII virus (MB085/05) suggests that it was maintained in the region as endemic infection.

In Malaysia, NDV has been isolated frequently from chickens and most of the isolates were belong to genotype VII viruses [18,32]. The MB061/06 isolate characterized in this study is the first genotype II NDV isolated from parrot in the peninsular Malaysia since it was reported in chicken in early 1980 s [33]. It is worth to point out that the grouping of isolate MB061/06 and LaSota under the same cluster in genotype II and the existence of similar C-terminus amino acid extensions length of HN protein suggested MB061/06 might be generated in nature from the LaSota vaccine strain.

## Conclusions

Our study indicates the occurrence of genotypes; II, VII and VIII and the presence of varied C-terminus extension lengths within Malaysian isolates. The deduced amino acid sequence of the F0 protein cleavage site showed also a unique amino acid motif in one of the isolates incriminated for sporadic cases occurred in different areas of the country. Approaches towards full characterization of isolates with unique F0 cleavage signal amino acid sequence and 6 amino acid extensions should be continued and intensified for deeper molecular knowledge and better intervention strategies.

## Methods

### Isolates

A total of eleven (11) NDV isolates, which were previously detected positive by real time RT-PCR between 2004 and 2007 at Biologics Laboratory, Faculty of Veterinary Medicine, Universiti Putra Malaysia (UPM) were employed in this study (Table 1). The virus isolates were propagated in the allantoic cavity of 9 days old specific-pathogen-free (SPF) embryonated chicken eggs according to European Community directive 92/66/EC [34] and identified by haemaglutination tests. Infected allantoic fluid samples were clarified by centrifugation and supernatant was stored at -70°C for later analysis.

### Viral RNA extraction

RNA extraction was performed from infected allantoic fluid using TRIzol^® ^Reagent (Invitrogen, USA) in accordance with the manufacturer's instructions. The RNA pellet was re-suspended in 20 μl nuclease-free water (Promega, USA) after air dried for immediate use or kept in -80°C for later use.

### Primers and reverse transcriptase-polymerase chain reaction (RT-PCR)

PCR amplification and sequencing were performed using previously described degenerative primers 5'-ATGGGC(C/T)CCAGA(C/T)CTTCTAC-3' (sense) and 5'-CTGCCACTGCTAGTTGTGATAATCC-3' (antisense) specific to fusion (F) protein gene [13] and HNNDV314 5'-ATATCCCGCAGTCGCATAAC-3'(sense) and HNNDV304 5'-TTTTTCTTAATCAAGTGACT-3'(antisense) specific to HN protein gene [35]. The primers generate an expected amplicon size of 535 bp (nt 47-535) fragment spanning the regions between nucleotides 47 and 581 of the fusion protein, which includes the F0 cleavage site and 320 bp products representing a fragment within HN protein gene, respectively. Standard RT-PCR was performed using Access^® ^One- step RT-PCR kit (Promega, USA) in a reaction volume of 25 μl. The cycling parameters for F gene specific primers were 48°C for 45 min at reverse transcription (RT), and 35 cycles of 94°C for 2 min, 56°C for 2 min, and 72°C for 1 min, followed by 72°C for 10 min and for HN gene specific primers; 48 min at 45°C for RT, 95 °C for 5 min, followed by 35 cycles of 1 min at 95°C, 1 min at 51°C and 1 min at 72°C and a final extension step at 72°C for 10 min (MyCycler^® ^Thermal Cycler, Bio-Rad, Hercules, CA, USA). The amplicons were separated by 1.5% agarose gel electrophoresis and visualized under ultraviolet after being stained with ethidium bromide.

### Sequencing of PCR products

Specific bands for each gene of interest were excised and purified by using GENEALL™Gel SV kit (General Biosystem, Inc., Korea) following the manufacturer's instructions. The purified DNA encoded for F and HN regions were sequenced by direct sequencing in both directions. Sequencing reactions were performed using ABI PRISM^® ^BigDye™ Terminator Cycle Sequencing Ready reaction kit v2.0 (Perkin Elmer, USA) in an automated DNA Sequencer (ABI PRISM^® ^377 DNA Sequencer). Each sample was sequenced three times to confirm consistency of the results.

### Nucleotide and deduced amino acid sequence analysis

Percent nucleotide identity and sequence editing were carried out using BioEdit software package version 7.01 [36]. Nucleotide analysis, prediction of amino acid sequences, and alignments were performed by Molecular Evolutionary Genetics Analysis, version 4.01 (MEGA 4) [37]. Determination of synonymous and non-synonymous substitution rates was conducted using the Nei-Gojobori method in Mega 4 [37]. Nucleotide sequences of partial F and HN gene of studied isolates were deposited in the GenBank and accession numbers are shown in Tables 4 and 5, respectively.

### Phylogenetic analysis

Phylogenetic analysis was carried out by comparing the variable portion of the F gene between 47 to 435 (389 bp) nucleotide sequences using the Clustal W multiple alignment method using MEGA 4 [37]. The phylogenetic tree was constructed using the neighbour-joining method after 1,000 bootstrap replicates. The sequence of the representative strains from each genotype and different geographical areas were retrieved from GenBank. Since no full-lengh sequence information at nt 47 to 435 was available for the remaining eight Malaysian isolates which were phylogenetically analysed in our previous study [18], sequences in this study were used. The accession numbers and country are as follows: AF2240 [AF048763], Malaysia; cockatoo/14698/90 [AY562985], Indonesia; Italien [EU293914], Italy; V4 Queensland [AF217084], Australia; Ulster/67 [AY562991], North Ireland; LaSota [DQ195265], USA; Miyadera [M18456], Japan; Herts/33 [AY741404], UK; CA1085/71 [AF001106], USA; H-10/72 [AF001107], Hungary; TX3503/04 [EU477190], USA; IraqAG68 [AF001108], Iraq; Ch/2000 [AF358788], China; TW/2000 [AF358786], Taiwan; TW/95-1 [AF083960], Taiwan; NDV05-055 [DQ439910], China; DK-1/95 [AF001129], Denmark; NDV05-027 [DQ439884], China; Q-GB 506/97 [AF109887], UK; QH-1/79 [AF378250], China; QH-4/85 [AF378252], China; ZhJ-1/85 [AF458023], China; FJ-1/85 [AF458009], China; ZA360/95 [AF109876], South Africa; ZW 3422/95 [AF109877], Zimbabwe; DE143/95 [AF109881], UK; Lebanon-70 [AF001110], Lebanon.

## Competing interests

The authors declare that they have no competing interests.

## Authors' contributions

ARO and AI designed and conceived the research, provided consultation and editing the manuscript. AB participated in the conceptual aspect of the work, performed the experiments and wrote the manuscript. MHB provided consultation and coordination. All authors read and approved the final manuscript
